# Validation and Assessment of a Posture Measurement System with Magneto-Inertial Measurement Units †

**DOI:** 10.3390/s21196610

**Published:** 2021-10-03

**Authors:** Davide Paloschi, Marco Bravi, Emiliano Schena, Sandra Miccinilli, Michelangelo Morrone, Silvia Sterzi, Paola Saccomandi, Carlo Massaroni

**Affiliations:** 1 Department of Mechanical Engineering, Politecnico di Milano, 20156 Milan, Italy; davide.paloschi@polimi.it; 2 Physical Medicine and Rehabilitative Unit, Università Campus Bio-Medico di Roma, 00128 Rome, Italy; m.bravi@unicampus.it (M.B.); s.miccinilli@unicampus.it (S.M.); m.morrone@unicampus.it (M.M.); s.sterzi@unicampus.it (S.S.); 3 Department of Engineering, Università Campus Bio-Medico di Roma, 00128 Roma, Italy; e.schena@unicampus.it (E.S.); c.massaroni@unicampus.it (C.M.)

**Keywords:** posture monitoring, inertial sensors, motion capture system, wearable systems, kyphosis, lordosis, range of movement

## Abstract

Inappropriate posture and the presence of spinal disorders require specific monitoring systems. In clinical settings, posture evaluation is commonly performed with visual observation, electrogoniometers or motion capture systems (MoCaps). Developing a measurement system that can be easily used also in non-structured environments would be highly beneficial for accurate posture monitoring. This work proposes a system based on three magneto-inertial measurement units (MIMU), placed on the backs of seventeen volunteers on the T3, T12 and S1 vertebrae. The reference system used for validation is a stereophotogrammetric motion capture system. The volunteers performed forward bending and sit-to-stand tests. The measured variables for identifying the posture were the kyphosis and the lordosis angles, as well as the range of movement (ROM) of the body segments. The comparison between MIMU and MoCap provided a maximum RMSE of 5.6° for the kyphosis and the lordosis angles. The average lumbo-pelvic contribution during forward bending (41.8 ± 8.6%) and the average lumbar ROM during sit-to-stand (31.8 ± 9.8° for sitting down, 29.6 ± 7.6° for standing up) obtained with the MIMU system agree with the literature. In conclusion, the MIMU system, which is wearable, inexpensive and easy to set up in non-structured environments, has been demonstrated to be effective in posture evaluation.

## 1. Introduction

The vertebral (spinal) column consists of thirty-three vertebral bony segments called vertebrae, divided into five regions: cervical, thoracic, lumbar, sacral and coccygeal segments. The spinal column presents a series of curvatures within the sagittal plane, which is defined as the plane running parallel to the sagittal suture of the skull, which divides the body into a left and a right section. These curvatures are either naturally convex anteriorly and concave posteriorly (thoracic kyphosis, sacrococcygeal kyphosis) or concave anteriorly and convex posteriorly (cervical lordosis, lumbar lordosis) and define the posture while standing [1]. An alternative definition of posture is the carriage and position of limbs or the body as a whole, indicating a certain feeling, pose, attitude or quality [2]. Improper posture can cause spinal deformations and can cause several complications during everyday life, such as lower back pain (LBP) and a reduction in kinematic capabilities [3]. Among the possible spinal deformations, scoliosis and hyperkyphosis are the most common in the thoracic region, whereas an abnormal increase in lumbar lordosis tends to generate excessive compression loads on the posterior elements of the spine [1].

To estimate the quality of a person’s posture, several approaches have been developed to monitor the thoracic kyphosis and the lumbar lordosis angles or the ranges of motion of the spinal regions. In clinical practice, visual observation is still largely adopted for assessing the condition of the patient [4,5] and is often enhanced by radiography tests that accurately present the shape of the spine [6]. The magnitude of spinal deformities can be derived from the radiography images with techniques such as the Cobb angle [7,8]. The Cobb angle identifies scoliosis in the frontal plane and measures kyphosis and lordosis in the sagittal plane. This method is highly reliable, but due to the X-ray dose delivered to the patient, other solutions, when available, should be preferred to radiography. From an external perspective, the spine’s health can be inferred by measuring the shape of the patient’s back. Typical methods include goniometers (both manual and electrical) and more hi-tech systems based on vision analysis. Electrical goniometers (electrogoniometers) measure angular rotations between the rigid extremities [9,10,11]. While these measurement systems are generally available and cost-effective, the main drawbacks are related to the difficulty of properly securing them to the body, the encumbrance of the device and the measurements limited to a single plane [9].

For these reasons, alternative measurement tools, such as those based on video (i.e., stereophotogrammetric motion capture systems such as MoCaps), have been largely used for posture monitoring in clinical settings. MoCaps can be used to record and analyze the motion of the body segments of a person: a set of calibrated infrared cameras tracks the spatial position of reflective markers that are positioned on specific body landmarks of the patient, and the data can be analyzed to derive relevant information [12]. Thus, MoCap systems are practical for monitoring dynamic activities. Moreover, they hold the benefit that several points of interest, defined through the passive markers, are located in sequential images and are then converted into real-space coordinates and used to infer the three-dimensional pose of the underlying body segments [13]. The main limitations of the MoCap system are related to the need to perform the measurements in a laboratory, where the cameras are calibrated, and to the need for dedicated personnel to post-process a massive amount of data [14]. Moreover, with the increase in the number of markers to be used, the time required to position them on the subject and post-process the data becomes large.

To guarantee the comfortable and long-term usability of the measurement system in the specific application, even without constant clinical supervision, a requirement of paramount importance is the wearability. For example, the control of posture in young athletes with LBP requires periodic assessments and monitoring for the rehabilitation process. This treatment can last for long periods of time; thus, the patients tend to resume an improper posture after a certain amount of time [15]. The examination is typically performed through static (e.g., by assessing deviations in the location of the center of pressure) and dynamic (e.g., by completing a movement task) evaluations [16]. Among the dynamic tests, forward bending [17,18] and sit-to-stand [19] are largely used to determine the health of the person’s spinal column. In this scenario, wearable devices for continuous posture monitoring and analysis can provide a quantitative measure of posture during everyday activities [20]. Inertial measurement units (IMU) are suitable for being embedded in smart garments and worn for long periods of time. They can combine the raw data and estimate their roll and pitch angle in space. IMU sensors with an embedded magnetometer are called MIMUs and can reliably measure also the yaw angle. The adoption of IMU and MIMU sensors is steadily growing also in the commercial field [21,22].

Other existing works propose the use of wearable inertial sensors for collecting postural information, starting from the analyses of trunk orientation and rotation during static and dynamic conditions [23,24]. With a similar rationale, inertial sensors are used in [25,26] to measure lumbar lordosis both in healthy subjects and in patients affected by LBP. These sensors are also very suitable for designing portable systems for healthcare applications, as proven by their long-standing use in biomechanical analyses [21]. A preliminary study on the feasibility of using MIMU sensors to measure the velocity profile of the seventh cervical vertebra (C7) is presented in [27].

The aim of this study is to develop an MIMU-based measurement system for both static and dynamic conditions for the measurement of the thoracic kyphosis and lumbar lordosis and to gain some preliminary insights into its capability to detect improper posture. The system is validated against a gold-standard instrument (i.e., a MoCap). Three MIMU sensors are placed on the bare backs of the volunteers in correspondence to the third thoracic vertebra (T3), the twelfth thoracic vertebra (T12) and the first sacral vertebra (S1), respectively. Reflective markers are placed on ten vertebrae, from the seventh cervical (C7) to the second sacral (S2), and their position in space is recorded with the MoCap system. The thoracic kyphosis and lumbar lordosis are calculated from the data of the motion capture system with the method presented in [28], whereas, for the MIMU sensors, the difference in slope between T3 and T12 (thoracic kyphosis) and the one between T12 and S1 (lumbar lordosis) is calculated. After the validation, the data from the inertial system are evaluated and analyzed from a clinical perspective.

## 2. Materials and Methods

Two measurement systems were used synchronously for assessing posture. The former is based on MIMUs, as previously mentioned, whereas the latter is a MoCap system. The MoCap system, which is largely used for non-invasive assessments [28,29,30], is validated only in static conditions for the calculation of the kyphosis and lordosis angles against CT images [31,32]. The MoCap system will therefore be used as a reference system for the validation of the MIMU system in static conditions. Subsequently, the MIMU system will be used for assessing the quality of the posture of the volunteers. The main characteristics of the systems are now presented.

### 2.1. Magneto-Inertial Measurement Units (MIMUs)

MIMUs are electronic boards containing a tri-axial accelerometer, gyroscope and magnetometer. These three units can independently measure inclination and rotations but are affected by limitations when used alone [33]. Regarding angle computations, accelerometers are reliable in quasi-static conditions, whereas gyroscopes are reliable in dynamic conditions. Lastly, magnetometers are biased by magnetic fields other than that of the Earth and disturbances in the surroundings. For these reasons, the data from these sensors are combined with sensor fusion techniques so that the information becomes accurate and reliable. The sensor fusion output can be either Euler angles (roll, pitch, yaw) or quaternions. Both representations are commonly used for expressing the orientation of a body or a frame in the three-dimensional space [33].

**Figure 1:**
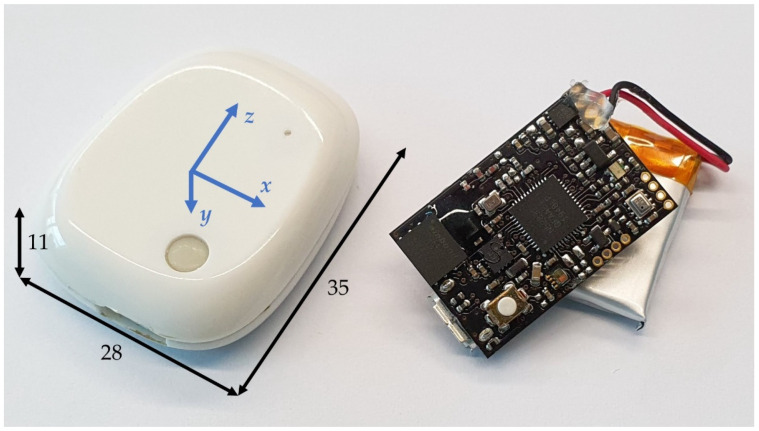
MetaMotionR. Encapsulated unit on the left with reference frame and dimensions in mm. Board and Li-Po battery on the right.

The specific MIMU used in the experimental trials is the MetaMotionR (MBIENTLAB INC, San Francisco, California, USA) [34]. MetaMotionR is an MIMU board with a lithium polymer (Li-Po) battery, a micro-controller for onboard elaborations and Bluetooth Low Energy (BLE) communication capabilities. The raw data from the accelerometer, gyroscope and magnetometer are transformed into quaternions by the onboard sensor fusion at a frequency of 100 Hz. The producer of the board reports an expected accuracy of <1° Root Mean Square (RMS) for the output of sensor fusion. The sensor is shown in Figure 1, where it is presented both with and without the protective case.

A quaternion is an angle representation given by the four components (w x y z). The orientation of a body is provided by a rotation (w) around an axis with components (x y z). Compared to Euler angles, the quaternions are numerically more stable and avoid the problem of gimbal lock. The gimbal lock is the loss of a degree of freedom (DoF) that occurs when using Euler angles to represent orientations, making them impractical in some applications [35].

The devices used in this study communicate with a computer via BLE and stream data continuously at a frequency of 100 Hz. Alternatively, the raw data can be stored in the internal memory of the sensors and downloaded later. The software used for the communication is MetaBase (MBIENTLAB INC, San Francisco, California, USA). The analysis of the MIMU data is performed after the data collection in the MATLAB environment and will be presented in Section 2.4.

### 2.2. Stereophotogrammetric Motion Capture System (MoCap)

Stereophotogrammetric MoCaps are instruments used in the field of biomechanics in a range of configurations and applications [36]. A MoCap typically consists of two or more cameras with infrared emitters that are able to track the trajectories of a number of photoreflective (active or passive) markers that are placed on a generic moving object [36,37]. When a marker is recorded from the cameras, the tridimensional trajectory can be retrieved from the planar camera frame with a resolution of more than 0.5 mm [12]. In clinical practice, MoCaps are used to investigate several aspects related to gait and posture [38,39,40], to indirectly estimate physiological parameters (e.g., breathing volumes [41,42]) from the 3D chest wall movements and to assess complex shape changes in various application scenarios [43,44].

Focusing on the posture analysis setup, cameras must be adequately installed in the dedicated room to surround the human body and be able to detect all the markers placed on body landmarks.

**Figure 2:**
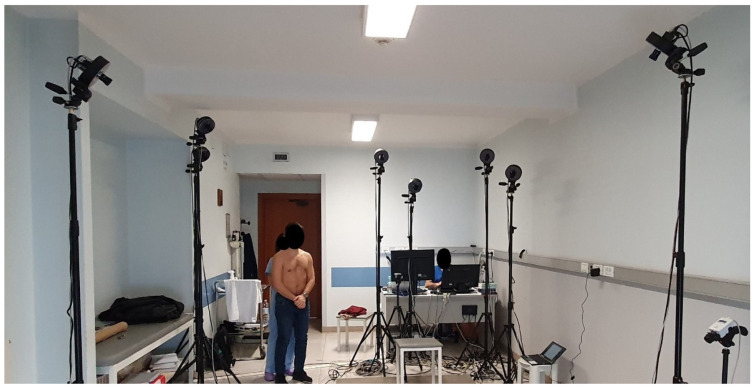
MoCap hardware setup. Infrared cameras are calibrated to track the markers’ trajectories in the dedicated volume. Reflective markers are applied to the back of the volunteer.

In our study, we used an eight-camera MoCap system (BTS D-Smart, by BTS Bioengineering S.r.l., Milan, Italy) and ten spherical photoreflective markers with a diameter of 12 mm (Figure 2). The raw 3D markers’ trajectories were recorded with BTS Tracker software (by BTS Bioengineering S.r.l., Milan, Italy) at a sampling rate of 60 Hz.

### 2.3. Patient Enrolment and Sensor Placement

Seventeen volunteers (seven women and ten men, age 35 ± 10 years, height 171 ± 9 cm, body mass 71 ± 13 kg) were enrolled in the study. All the tests were carried out in compliance with the ethical approval (09/19 OSS ComEt Università Campus Bio-Medico di Roma UCBM), and, prior to the tests, all the participants provided their informed consent. One experienced physiotherapist (M.B.) placed the MIMU sensors and the markers on the backs of the volunteers.

To establish the number and the position of the sensors and markers on the spine for back posture monitoring, we followed the indications of previous related studies [23,28,29,45], where the local geometry of portions of the backs of the subjects are used for inferring the magnitude of kyphosis and/or lordosis. With a similar rationale to the studies mentioned above, and with the knowledge acquired in the previous work of our group on posture monitoring [27], the positions of three MIMU sensors along the spine were chosen to be T3, T12 and S1. The locations of the ten reflective markers for the MoCap system were selected as C7, T2, T4, T5, T6, T8, L1, L3, L5, S2.

**Figure 3:**
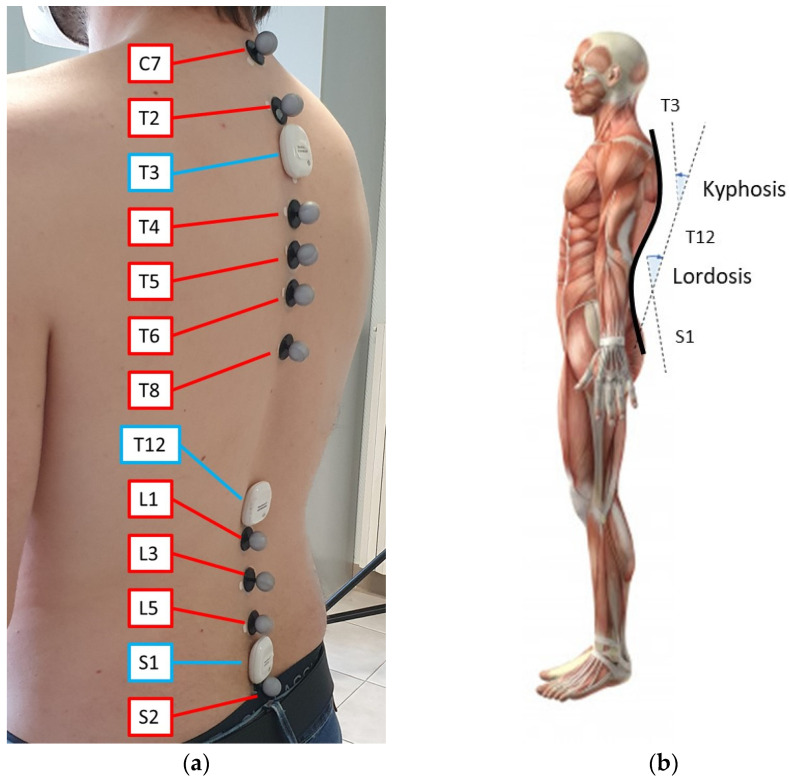
Experimental setup. (**a**) MIMU locations are indicated in blue, MoCap markers in red; (**b**) Scheme of kyphosis and lordosis. The angles calculated in the sagittal plane with the MIMU sensors at T3, T12 and S1 are indicated by dashed lines.

The locations of the MIMU sensors and MoCap markers on the back of one of the volunteers are shown in Figure 3a. Regarding the measurement performed with the MIMU sensors, the kyphosis and lordosis angles are evaluated as the difference in the angle between T3 and T12 (kyphosis angle) and between T12 and S1 (lordosis angle), as shown in Figure 3b. The kyphosis and lordosis angles can be identified with the MoCap system in static conditions [28] by interpolating the positions of the markers with a 5^th^ order polynomial and then finding the convexity changes in its gradient. Details of the calculation of the angles and data analysis are provided in the next section.

### 2.4. Calculation of Kyphosis and Lordosis Angles

The measurement of the kyphosis and lordosis angles was performed with both MIMU and MoCap systems, following two different procedures, as described hereafter.

The quaternion (*w x y z*) acquired from the MIMU sensor can be used in the following equation to retrieve the associated rotation matrix:

(1) $$R=\left[\begin{array}{ccc} 1-2y^{2}-2z^{2} & 2\left(xy-zw\right) & 2\left(xz+yw\right)\\ 2\left(xy+zw\right) & 1-2x^{2}-2z^{2} & 2\left(yz-xw\right)\\ 2\left(xz-yw\right) & 2\left(yz+xw\right) & 1-2x^{2}-2y^{2} \end{array}\right],$$

where *w*, *x*, *y* and *z* are the components of the quaternion and R is the associated rotation matrix. The rotation matrix has unitary norm, and each column describes the components of the three axes of a frame (*x*’, *y*’, *z*’). The angle between the vertical axis *z*’ and the reference axis [0 0 1]’ is the pitch angle and was used to evaluate the change in posture of the subjects. The kyphosis and lordosis angles assessed with MIMU sensors were calculated as the difference in the pitch angle between the sensor in T3 and the one in T12 (kyphosis angle), and between the sensor in T12 and the one in S1 (lordosis angle).

**Figure 4:**
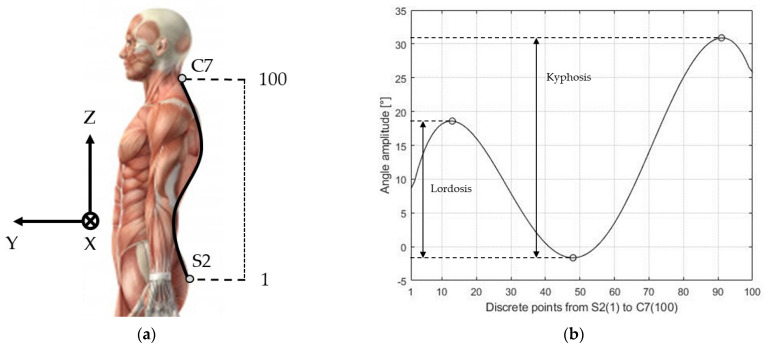
MoCap polynomial method. (**a**) Discretization of the spine from the first marker (S2) to the last marker (C7) and reference triplet XYZ; (**b**) Local angle at each discrete point. The horizontal axis represents the discrete locations between the first marker on S2 and the last one on C7. The vertical axis represents the local slope in degrees of the considered location.

The data acquired from the MoCap system were three-dimensional coordinates (expressed in mm). The space dimension was reduced to the plane in which the movement occurred, so lateral bending was ignored. The decision regarding which plane was to be considered for the analysis depended on the nature of the exercise itself. The coordinates of the reflective markers were used to calculate a polynomial that described the shape of the back of the subject. As proposed by Ranavolo and colleagues [28], the minimum order that is greater than four (to obtain two convexity changes) and that achieves an R^2^ greater than 0.99 when compared to full-spine digitized radiographs in the reconstruction of the anatomical spinal curve in the sagittal plane is five. In this regard, we chose a 5^th^ order polynomial to represent the back shape of the subjects involved in our study. The polynomial was discretized on 100 points from S2 to C7, to obtain a smooth function describing the shape of the back of each volunteer (Figure 4a). The gradient of the curve was then derived and inspected for the changes in convexity. Ideally, the changes in convexity were three, located in the sacral, thoracic and cervical regions, respectively. The angle calculated with this method is shown in Figure 4b.

The difference in slope between the cervical and the thoracic regions is the kyphosis angle, whereas the difference between the thoracic and sacral region is the lordosis angle (Figure 4b).

### 2.5. Experimental Protocol and Data Analysis

Each volunteer was asked to perform a forward bending and a sit-to-stand trial, two times each. Within a single exercise, the task was repeated ten times.

To validate the MIMU-based measuring system, the thoracic kyphosis and the lumbar lordosis parameters were compared between the MIMUs and the MoCap system. The comparison between the MIMU and the MoCap angles was carried out at every static phase of the exercises during which the subject was standing still and had not yet started the next repetition. The comparison between the two systems in the stationary phase was performed since the MoCap system is validated for this calculation only in static conditions [28], so it is calculated every time the subject returns to an upright standing position. The root mean square errors (RMSE), the average values and the standard deviations were then calculated for each volunteer and for each exercise (i.e., forward bending and sit-to-stand). The RMSE was calculated as follows:

(2) $$RMSE=\sqrt{\frac{1}{n}\sum_{i=1}^{9}\left(\vartheta_{MIMU,i}-\vartheta_{MoCap,i}\right)},$$

where $\vartheta_{i}$ is the i-th measurement of either the kyphosis or lordosis angle performed by the MIMU or the MoCap system for nine repetitions during the forward bending and sit-to-stand tests, excluding the tenth. The last repetition was excluded because the candidate assumed a different position when the task was over, and he/she could relax, thus increasing the dispersion of the kyphosis and lordosis values. After this validation study, the MIMU data were analyzed to retrieve the range of movement (ROM) of the forward bending and sit-to-stand exercises. The ROM is a lumbo-pelvic kinematic characteristic and one of the basic components of the physical examination of people with LBP [3]. The ROM was calculated as the angle difference that every sensor measured from the starting upright position to the most bent position. To consider only the contribution of each location, the angles measured by sensors in the lower position were subtracted from the ones in the upper locations. In particular, the ROM of T3 was the absolute measure of T3 minus the absolute measure of T12, and the ROM of T12 was the absolute measure of T12 minus the absolute measure of S1. The absolute displacement of S1 coincided with its ROM. During the forward bending test, the percentual ratio of S1 on T12 (called lumbo-pelvic rhythm) was analyzed and compared to a similar study [46]. The ROM during the sit-to-stand exercise was evaluated both during the sitting down phase and during the standing up phase.

## 3. Results

### 3.1. Comparison of the Two Measurement Systems

**Figure 5:**
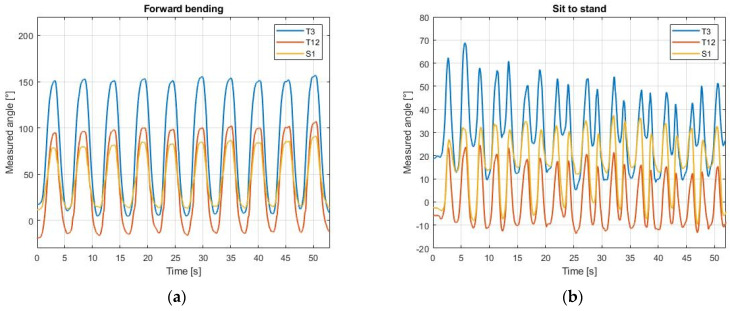
Inclination from the vertical measured by MIMUs. The angles obtained from each sensor are positive for a forward inclination and negative for a backward inclination. (**a**) Angles during forward bending; (**b**) Angles during sit-to-stand.

An exemplary time evolution of the angles in T3, T12 and S1 measured with the MIMU system is shown in Figure 5 for the forward bending and the sit-to-stand exercises recorded from one volunteer.

**Figure 6:**
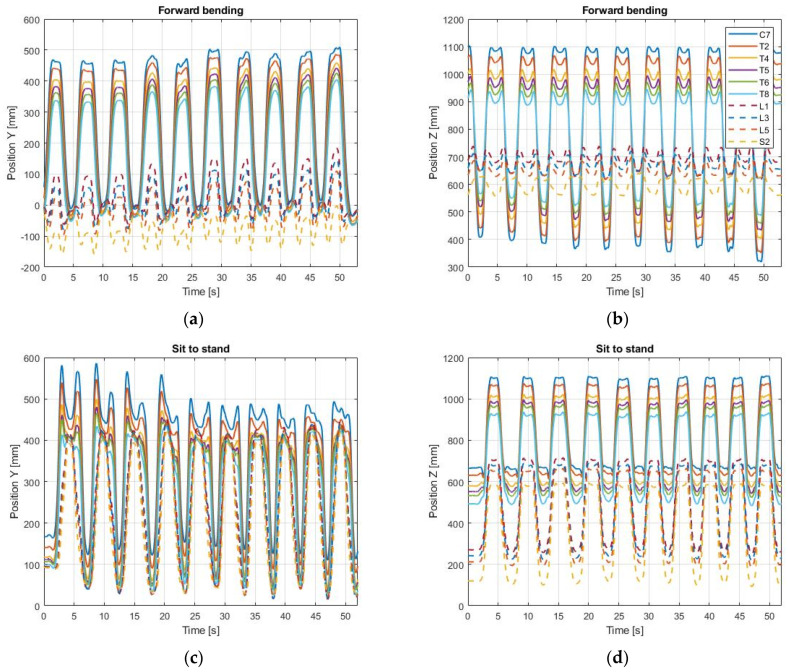
MoCap data. The position of every marker is shown in the sagittal plane, defined by the Y and Z axes of the calibration frame. (**a**) Coordinates in the Y axis during forward bending; (**b**) Coordinates in the Z axis during forward bending; (**c**) Coordinates in the Y axis during sit-to-stand; (**d**) Coordinates in the Z axis during sit-to-stand.

The output of the MoCap system were the three-dimensional coordinates of every reflective marker, relative to the position of the calibration frame. The data for one subject are shown in Figure 6 for the forward bending and the sit-to-stand exercises, where the lateral movement along the X axis is negligible compared to the sagittal movement in the Y–Z plane.

From the results of Figure 6, the kyphosis and lordosis angles were calculated with the polynomial method (Section 2.4) only when the volunteer was standing still between the repetitions. For the forward bending, this static position corresponds to the plateaus of the markers C7-T8 in Figure 6b. For the sit-to-stand, this condition corresponds to the plateaus of the markers C7-T8 in Figure 6d.

**Figure 7:**
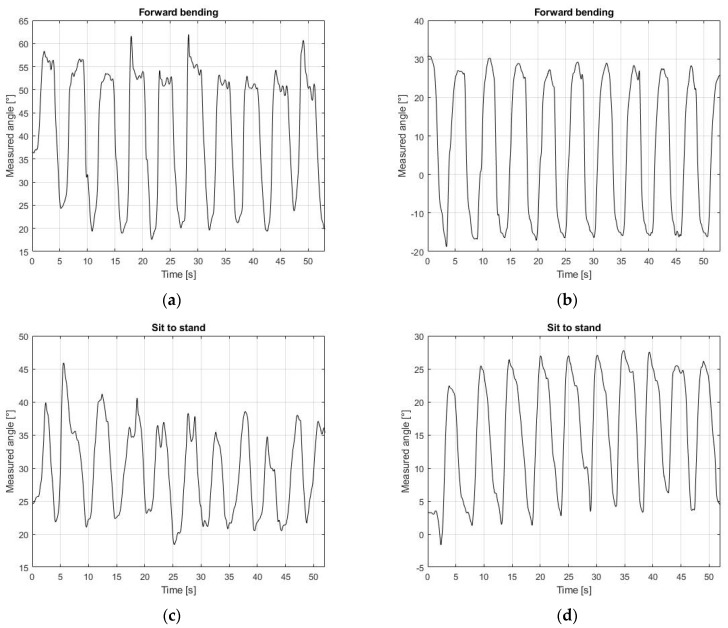
Thoracic kyphosis and lumbar lordosis. The difference in amplitude between the MIMU sensors represents the thoracic kyphosis (T3-T12) and lumbar lordosis (S1-T12). (**a**) Kyphosis angle during forward bending; (**b**) Lordosis angle during forward bending; (**c**) Kyphosis angle during sit-to-stand; (**d**) Lordosis angle during sit-to-stand.

Conversely, the MIMU sensors could measure the time evolutions of the thoracic kyphosis and lumbar lordosis as the difference between the values of T3-T12 and S1-T12, respectively. These results are shown in Figure 7.

**Figure 8:**
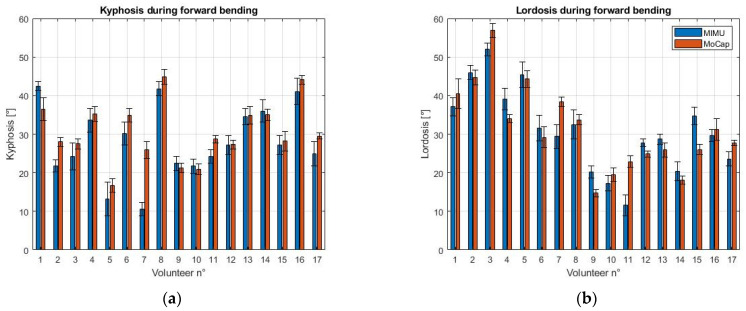

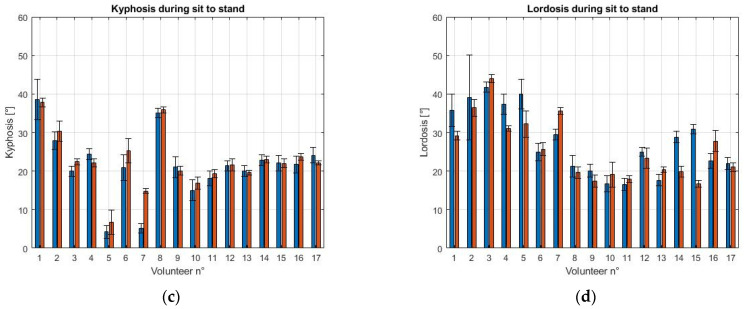
Comparison of the measurement systems. The average MIMU angle is reported in blue, the average MoCap angle is reported in red. (**a**) Kyphosis during forward bending; (**b**) Lordosis during forward bending; (**c**) Kyphosis during sit-to-stand; (**d**) Lordosis during sit-to-stand.

The thoracic kyphosis and lumbar lordosis were calculated from the MoCap data with the polynomial method presented in Section 2.4. The results for the average kyphosis and lordosis values for each volunteer and for each exercise (i.e., forward bending and sit-to-stand), obtained with the MIMU and the MoCap systems, are presented in Figure 8. 

The RMSE, the mean difference (calculated as the average of the differences in the mean values of the two measurement systems, across the 17 volunteers) and standard deviation of the difference between the MIMU and the MoCap systems are reported in Table 1.

**Figure 9:**
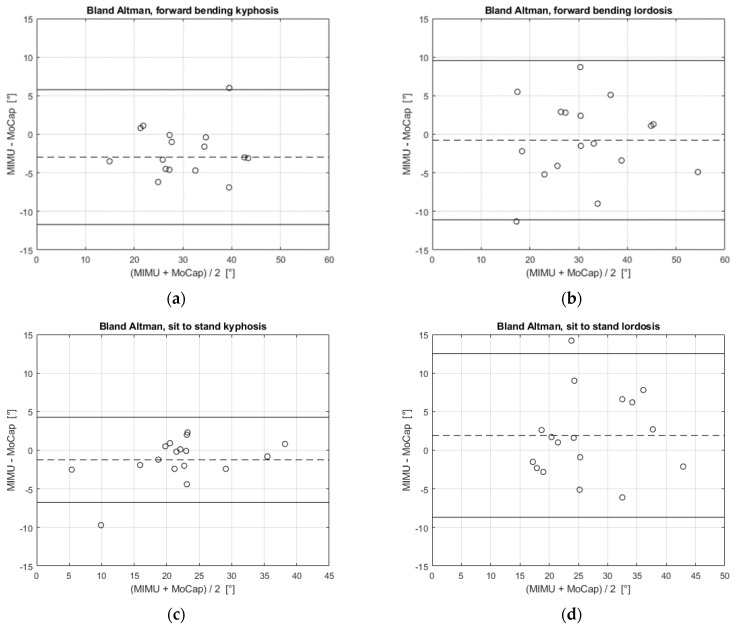
Bland–Altman test. Each of the 17 subjects is represented by the average of the kyphosis or lordosis angle accordingly and is plotted as a hollow circle in the graphs. The MoD is the dashed line, the LoA are the continuous lines. (**a**) Kyphosis angle during forward bending; (**b**) Lordosis angle during forward bending; (**c**) Kyphosis angle during sit-to-stand; (**d**) Lordosis angle during sit-to-stand.

Bland–Altman analysis was performed for the statistical investigation of the agreement between the two measuring systems. The test considers the difference between the values measured by each system and plots them against the average of the measurements of both systems. The two parameters that were considered in the Bland–Altman analysis were the limits of agreement (LoA) and the mean of differences (MoD). The LoA represents the agreement interval, within which 95% of the differences in the data measured with the MIMU system, compared to the MoCap system, fall. The LoA were calculated as MoD ± 1.96 SD, where SD is the standard deviation of the differences. The results for the Bland–Altman tests obtained for the mean values of the kyphosis and lordosis of the 17 volunteers (shown in Figure 8) are shown in Figure 9.

The MoD and LoA values retrieved from the Bland–Altman analysis are reported in Table 2, for each exercise.

From Figure 9, we can observe that the difference between the two systems was not dependent on the magnitude of the angle (e.g., the difference did not increase with the angle). Moreover, the results in Table 2 show that the MIMU system slightly underestimated the kyphosis angle (MoD is negative), for both the forward bending and sit-to-stand tasks. Instead, the lordosis angle was slightly underestimated in the forward bending exercise (MoD = −0.8°) and overestimated in the sit-to-stand task (MoD = 1.9°). Additionally, LoA values for the lordosis angle were larger than LoA for kyphosis in both exercises (9.6/−11.1° and 12.5/−8.7° for lordosis vs. 5.8/−11.7° and 4.3/−6.7° for kyphosis).

### 3.2. Assessment of the Range of Movement (ROM)

Once the MIMU-based system was validated against the MoCap system, it was used to assess the ROM for the two exercises, as described in Section 2.5. The ROM was calculated for all the subjects during the forward bending test, and every time the subject was standing up or sitting down during the sit-to-stand test.

**Figure 10:**
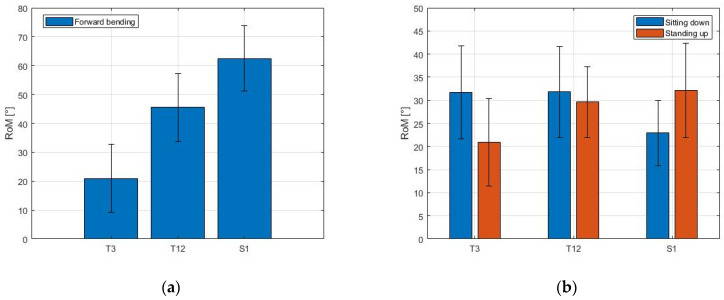
Range of movement (ROM) measured with MIMUs. The ROM of each sensor numerically shows the contributions of the three segments during movement. (**a**) Forward bending; (**b**) Sit-to-stand.

The contributions of the three body areas during movement are given by the MIMU sensors as the ROM of T3, T12 and S1. The average value among all volunteers is shown in Figure 10 for each exercise.

The ROM increased along the spine during the forward bending, from T3 to S1 (see Figure 10a). During the sit-to-stand exercise, the ROM of the three zones exhibited a different behavior when considering the sitting down and the standing up phases (Figure 10b). During the sitting down phase, the ROM of T3 and T12 was similar (31.7 ± 10.0° and 31.8 ± 9.8° for T3 and T12, respectively) and decreased in correspondence with S1 (mean value 22.9 ± 7.1°). During the standing up phase, the ROM of T3 was 20.9 ± 9.5° and increased in correspondence with T12 (29.6 ± 7.6°) and S1 (32.1 ± 10.2°).

**Figure 11:**
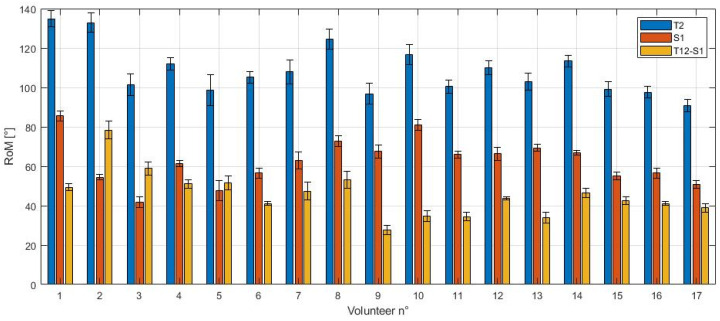
Lower contributions to forward bending. The average peak contributions of T12 and S1 are shown for each volunteer for the forward bending test. The difference between the two sensors represents the lumbar contribution.

Figure 11 shows the different contributions of the thoracic (T12) and sacral (S1) sections. The difference between the two values (T12-S1) is the lumbar contribution.

The peak value of T12 ranged from 90.8° to 134.8°, with a maximum standard deviation within the same subject of 7.9°. The peak value of S1, which coincided with its ROM, ranged from 41.7° to 85.5°, with a maximum standard deviation of 5.1°. The ROM of T12, given by the difference between the peak value of T12 and S1, is also called the lumbar contribution. Its values ranged from 27.7° to 78.4°, with a maximum standard deviation of 4.5°, and it was almost always lower than the pelvic contribution (S1). The average percentual contribution between lumbar and peak thoracic angle (i.e., the lumbo-pelvic rhythm) was calculated as the percentual ratio between T12-S1 (yellow bar) and T12 (blue bar). It was equal to 41.8 ± 8.6%.

## 4. Discussion

In this work, we propose a measurement system based on the use of three MIMU sensors for estimating both the thoracic kyphosis and the lumbar lordosis and for the evaluation of posture. The main results of the study are the validation of the proposed MIMU system against the MoCap system, used as a reference during two well-established clinical exercises (i.e., sit-to-stand and forward bending), the estimation of thoracic kyphosis and the lumbar lordosis angles and the calculation of the lumbo-pelvic RoM from MIMU data. The sensors were worn by seventeen volunteers who performed typical exercises in the field of spinal posture analysis.

The study on the validation of the MIMU system shows that the kyphosis and lordosis calculation carried out with the MIMUs is compatible with that performed with the MoCap system, as revealed by the Bland–Altman analysis shown in Figure 9. This result proves the feasibility of the use of the MIMU system, which is lightweight and inexpensive, instead of the MoCap system. The evaluation of the kyphosis angle shows better compatibility since the LoA of the Bland–Altman analysis are narrower than those for the lordosis angle (5.8/−11.7° and 4.3/−6.7° for kyphosis vs. 9.6/−11.1° and 12.5/−8.7° for lordosis). Although the MIMU and MoCap systems present large differences for some volunteers, the average difference between them is less than 4° for the kyphosis and less than 2° for the lordosis (Table 1). In our case, the higher average difference for the kyphosis angle is also due to the data collected for volunteer 7, where the mismatch is more important (Figure 8). The greatest difference between the two systems in the calculation of the lordosis angle is represented by volunteer 15, both in the forward bending and sit-to-stand tasks. This outcome is probably due to either the non-idoneous placement of a MIMU sensor on the back of this subject, or directly to the anatomy of the volunteer, for which the application of the sensors in correspondence with the T12 and S1 vertebrae may not be representative of the lumbar lordosis. Although the inclusion of these two volunteers in the analysis affected the evaluation of the performance of the measurement system, satisfactory results were achieved. On the other hand, the polynomial method used for the MoCap data, even though already validated, is not always applicable. This happens because, depending on the shape of the subject’s back, the convexity changes in the gradient might not be present, as suggested in [28]. Although the shape of the spine is monitored also in dynamic conditions with the MoCap system, the calculation of the thoracic kyphosis and lumbar lordosis cannot be carried out if the convexity changes are not present in the polynomial reconstruction. The MIMU sensors can be instead placed in the exact location of the spinous processes T3, T12 and S1. For this reason, the MIMU system is more adaptable to the anatomy of the subject [27] and has no ambiguity regarding the angles that it monitors. Furthermore, the MIMU sensors allow us to dynamically measure the kyphosis and lordosis angles, whereas the polynomial method employed for the MoCap system is validated only for static conditions.

The RMSE of the calculation of the kyphosis and lordosis angles across all volunteers ranged from 3.0° to 5.6°, as reported in Table 1. These results are in line with other studies that use MoCap as a reference system and MIMUs for evaluating the motion of body segments during physical exercises [47,48], where an RMSE < 5° was obtained. The kyphosis and the lordosis angles computed in this work were derived as the difference between two values (T3-T12 and T12-S1), whereas the aforementioned studies calculated the RMSE of single inclinations of the back, where a lower error was expected.

The MIMU data can also be used to analyze the contributions of different segments of the spine during movement. During forward bending, the sacral part of the spine carries out most of the bending, as shown in Figure 10a. The results obtained—specifically the average lumbo-pelvic rhythm of the volunteers, estimated to be 41.8 ± 8.6%—are comparable with [46], where MIMU sensors were placed in T12 and S2. During sit-to-stand (Figure 10b), the lower thoracic (T12) contribution remained constant, whereas the upper thoracic (T3) and sacral (S1) contributions depended on whether the volunteer was sitting down or standing up. The average lumbar contribution that was measured during sit-to-stand was 31.8° and 29.6°, for the sitting down and standing up phase, respectively. This result agrees with other studies on lumbar mobility during sit-to-stand [49], where the average value of lumbar ROM for healthy volunteers was reported to be equal to 32.07 ± 6.77°.

ROM assessment is commonly used by clinicians to assess patients with LBP, to identify any dysfunctional patterns and to monitor changes after medical and/or rehabilitation treatments [50]. In fact, patients with LBP usually show an average reduction in flexion ROM compared to a group of subjects without LBP [3]. The system proposed in this study is easy to use in the clinical setting, offering a more precise and detailed evaluation than what is commonly obtained using the standard tools of outpatient settings, such as goniometers. The use of three MIMU sensors allows not only the assessment of the kyphosis and lordosis of the subject (static and dynamic) but also the evaluation of the mobility of the spinal segments that are being monitored and yields results comparable to other studies in the same field [49]. Further development of this system could provide other useful information, such as movement speed, fluidity and proprioception, allowing clinicians to better understand which aspects of movement are altered. It is, in fact, necessary to have easy-to-use tools that support the clinician in carrying out a more accurate classification of lower back problems [51] and in prescribing a personalized and specific treatment with potential benefits in terms of direct and indirect socio-health-related costs.

In agreement with the observations of several recent studies, in our work, MIMUs also hold the advantage of being portable and easily installed and worn by the subjects. On the other hand, the MoCap system presents some disadvantages, such as the ample and dedicated space for the tests, the several cameras to set up and calibrate and the time-consuming manual post-processing needed due to the occlusions of markers during the exercise [47]. Regarding the proposed system, limitations related to hardware and communication should also be considered for its efficient use. Specifically, the device handling all the data (i.e., a computer) must ensure reliable and stable communication with the sensors via BLE. This requirement becomes critical for applications requiring a greater number of sensors, such as the simultaneous monitoring of multiple physiological parameters. Furthermore, the communication should enforce adequate security protocols for privacy reasons [52]. Depending on the number of operations carried out by each sensor, a properly dimensioned Li-Po battery should be chosen to ensure that the device remains operational for a sufficient number of hours, without being too bulky.

In conclusion, the main novelty of the proposed MIMU-based system over other systems [21,46] regards the possibility to dynamically measure both the thoracic kyphosis and the lumbar lordosis angles and the ROM of two segments, thanks to the use of three sensors. Differently from other studies, which essentially monitor the acceleration of body segments due to posture change [53], the measurement approach here implemented allowed the identification of relevant angles from a clinical viewpoint (i.e., the kyphosis and lordosis angles). Since the values of these angles, along with the ROM, are commonly used by physicians to assess the posture of patients, the proposed system can provide valid support in the monitoring of significant posture-related parameters quantitatively and continuously.

In the future, the results of this study should be extended to a larger population of volunteers, which should also include patients with LBP. This step will permit assessment of the proposed MIMU system in a clinical setting and its validation to estimate the quality of the posture of patients during standard clinical protocols for diagnosis and treatment.

## 5. Conclusions

The study presents a system for spinal evaluation based on MIMU sensors and validates it against a MoCap system, which was selected as the gold standard. Three inertial sensors were selected for calculating the thoracic kyphosis and lumbar lordosis angles, but the number can be increased for a more precise segmentation of the spine. Although some differences in terms of accuracy exist between the MIMU and the MoCap system, the paramount features of the proposed system are the wearability, the low cost and the ease of use. The data analysis is more straightforward than that of the MoCap system, as the output of the MIMU sensor fusion is already an angle. On the other hand, improper positioning of the MIMU sensors leads to erroneous measurements. This risk may be mitigated by using a custom-designed garment for unsupervised use that ensures the correct positioning of the sensors at all times. A limitation of the current work is the relatively small number of volunteers, so that a statistical clinical approach cannot be yet fully pursued.

Future works will consider a large sample of volunteers, with both healthy subjects and patients with relevant LBP, and will aim at numerically estimating the state of health of the subject.

## Figures and Tables

**Table 1:** RMSE, mean difference and standard deviation in the calculation of kyphosis and lordosis (MIMU and MoCap).

Dataset	RMSE (°)	Mean Difference (°)	Standard Deviation (°)
Kyphosis—forward bending	5.0	−3.6	4.9
Lordosis—forward bending	5.0	−1.4	5.5
Kyphosis—sit-to-stand	3.0	−1.3	2.8
Lordosis—sit-to-stand	5.6	1.9	5.4

**Table 2:** Bland–Altman test in the calculation of kyphosis and lordosis (MIMU and MoCap).

Dataset	MoD [°]	LoA [°]
Kyphosis—forward bending	−3.0	5.8/−11.7
Lordosis—forward bending	−0.8	9.6/−11.1
Kyphosis—sit-to-stand	−1.2	4.3/−6.7
Lordosis—sit-to-stand	1.9	12.5/−8.7

## Data Availability

The data presented in this study are available on request from the corresponding author. The data are not publicly available due to privacy restrictions.
